# Lack of effect of Imrecoxib, an innovative and moderate COX-2 inhibitor, on pharmacokinetics and pharmacodynamics of warfarin in healthy volunteers

**DOI:** 10.1038/s41598-019-51755-z

**Published:** 2019-10-31

**Authors:** Yani Liu, Rui Zhang, Zhongfang Li, Jiali Zhou, Tingyu Yang, Chunxiao Yang, Xixi Huang, Yu Zhang, Shaojun Shi

**Affiliations:** 1 Department of Pharmacy, Union Hospital, Tongji Medical College, Huazhong University of Science and Technology, Wuhan, 430022 China; 2 Clinical Research Organization for Pharmaceutical Products, Union Hospital, Tongji Medical College, Huazhong University of Science and Technology, Wuhan, 430022 China

**Keywords:** Toxicology, Adaptive clinical trial

## Abstract

Imrecoxib is a registered treatment for osteoarthritis pain symptoms in China. This study aims to assess the effect of imrecoxib on the pharmacodynamics and pharmacokinetics of warfarin. 12 healthy male volunteers with CYP2C9*3 AA and VKORC1 AA genotypes took a 5 mg dose of warfarin both alone and concomitantly with steady-state imrecoxib. Both warfarin alone and concomitantly with imrecoxib have safey and good tolerance across the trial. Following warfarin and imrecoxib co-administration, neither C_max_, AUC_0-t_ and t_1/2_ of warfarin enantiomers nor AUC of international normalized ratio (INR) were markedly different from those of warfarin alone. The geometric mean ratios (GMRs) (warfarin + imrecoxib: warfarin alone) of INR_(AUC)_ was 1 (0.99, 1.01). The GMRs of warfarin AUC_0-∞_ (90% confidence interval, CIs) for warfarin + imrecoxib: warfarin alone were 1.12 (1.08, 1.16) for R-warfarin and 1.13 (1.07, 1.18) for S- warfarin. The 90% CIs of the GMRs of AUC_0-∞,_ C_max_ and INR _(AUC)_ were all within a 0.8–1.25 interval. The combination of warfarin and imrecoxib did not impact the pharmacodynamics and pharmacokinetics of single-dose warfarin; therefore, when treating a patient with imrecoxib and warfarin, it is not required to adjust the dosage of warfarin.

## Introduction

The incidence of Osteoarthritis (OA) is more than 50% in people over 60 years old in China^1,2^. OA has a serious impact on patients’ ability to function and leads to considerable societal costs^3^. The clinical characteristics of OA are related to the development of aches, discomfort, rigidity, cartilage degradation and bone remodeling^1^, OA’s treatment focuses on symptom control, and mainly aims to relieve joint swelling and ease pain. Selective cyclooxygenase (COX)-2 inhibitor are frequently prescribed to OA patients due to their inhibition of the inflammatory cascades and relief of the pain symptoms.

**Figure 1:**
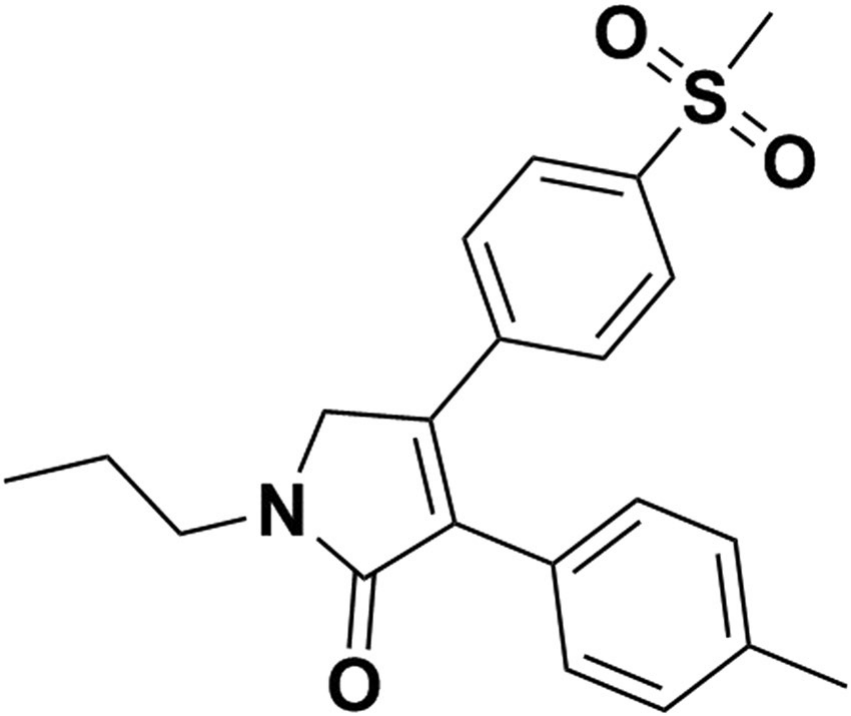
Chemical structure of Imrecoxib (4-(4-methylsulfonyl-phenyl)-1-propyl-3-(p-tolyl)-1H-pyrrol-2(5 H)-one).

Imrecoxib, 4-(4-methylsulfonyl-phenyl)-1-propyl-3-(p-tolyl)-1H-pyrrol-2(5H)-one (Fig. 1), is a new and moderate selective COX-2 inhibitor^4^. It is currently registered in China for the symptomatic treatment of osteoarthritis and has been widely prescribed since its launch in 2011^5^. It has been reported that the single-dose pharmacokinetics of imrecoxib were linear over the 30 to 200 mg dose range. The t_1/2_ of imrecoxib is 20 hours, t_max_ occurred at 2 hours following oral consumption. No accumulated effects were observed in plasma after administration of 200 mg imrecoxib, bid, for 11 consecutive days^6^. Imrecoxib is metabolized by hepatic isoenzyme CYP2C9, 2D6 and 3A4 enzymes, with rates of 62.5%, 21.1% and 16.4%, respectively. Following oral ingestion, the 4′-methyl group of imrecoxib is hydroxylized to the 4′-hydroxymethyl metabolite by CYP2C9, and further oxidized to 4′-carboxylic acid metabolite^7^. The main metabolites in urine are the hydroxymethyl and carboxy metabolites produced by the oxidation of phenylcyclomethyl, while the carboxylic acid metabolite is primarily excreted from feces^8^.

Warfarin is effective for preventing intravenous thromboembolism, cardiovascular and cerebrovascular infarction, and other thromboembolic disorders. It is a racemic mixture of two isomers, CYP2C9 enzyme metabolizes S-warfarin, and CYP1A2 and CYP3A4 are responsible for metabolism of R-warfarin^9^, which make it susceptible to interaction with numerous inhibitors and inducers of CYPs. This interaction might lead to either an inability to achieve the expected anticoagulant effects or an enhanced bleeding risk induced by excessive anticoagulation^10^. In a US retrospective prescription analysis, nonsteroidal anti-inflammatory drugs (NSAIDs) with warfarin was the most frequently occurring medication pair in drug-drug interactions (DDI) reports^8^, and 24% of warfarin recipients would be given NSAIDs treatment within two years^8,11^. NSAIDs impair the gastrointestinal mucosa and aggregation of platelets by inhibiting the COX-1 isozyme^12,13^, which significantly enhances the risks of hemorrhage in patients taking warfarin^14–17^. Specific inhibitors of COX-2 have been approved for OA therapy. COX-2 specific inhibitors do not cause severe bleeding and are thus considered potentially safe for warfarin-treated patients^18^. However, increasing evidences of myocardial infarction, as well as cardiovascular secondary action relate to COX-2 specific inhibitors, such as rofecoxib and valdecoxib, lead to their retreat from the market^19,20^. Therefore, a moderate COX-2 selective inhibitor with decreased bleeding risk than NSAIDs and reduced cardiovascular secondary action compared with COX-2 specific inhibitors, would be appropriate for management of OA.

Clinical trials have demonstrated that imrecoxib shows 50% inhibitory concentration (IC_50_) of COX-1 and COX-2 isozymes by 115 ± 28 nmol/L and 18 ± 4 nmol/L, respectively^5^. The selective index (IC_50_, _COX-1/COX2_) was 6.39, which was between that of meloxicam and celecoxib^21^. From a clinical perspective, whilst the lack of pharmacokinetic and pharmacodynamics effects on warfarin are important in terms of dose adjustment etc, the risk of bleeding due to GI irritation is still significant with NSAIDs (including a drug of relative COX-2 specificity) plus warfarin, particularly in the elderly. In addition, both S-warfarin, the more potent enantiomer of warfarin, and imrecoxib are metabolised by the CYP2C9 enzyme^8,22^. However, whether co-administration of imrecoxib and warfarin would result in DDI was not investigated. In this study, we evaluated the potential DDI of imrecoxib and warfarin by comparing the pharmacodynamic and pharmacokinetic parameters of warfarin with and without co-administration of imreocxib in healthy male volunteers. We also tested the safety and tolerability of study drugs across the trial^23^.

## Methods

### Ethics

Current study was conducted in conformity to the Declaration of Helsinki (as revised in Brazil, 2013)^24^, Good Clinical Practice (GCP) guidelines of China Food and Drug Administration (CFDA)^25^ and the technical guidelines for clinical pharmacokinetic study of chemical drugs^26^. CFDA (no. 2011S00434) and the independent ethics committee (Tongji Medical College, Huazhong University of Science and Technology, no. (2014)185] reviewed and approved this study protocol. Written informed consent was required for every volunteer before any study procedures^27^.

### Subjects

Twelve subjects were enrolled in this study. The inclusion requirements were (i) male; (ii) BMI ranged from 19 to 24 kg ⁄m^2,^^26^; (iii) aged between 18 to 40; (iv) qualified for complete health examination, including vital signs, electrocardiograms, routine blood test, urinalysis, biochemistry laboratory parameters, chest X-ray, liver and renal function tests are normal or not clinical significantly abnormal. (v) a condition of normal coagulation function (prothrombin time - PT, INR and fibrinogen) and negative serological test (HBsAg, HCV and HIV antibodies); (vi) voluntary signing of informed consent forms^27^.

As we previous reported^27^, subject would be excluded if he met these criterions: (i) hypersensitivity or allergy to the study drugs; (ii) any diseases or unstable medical history that may disturb the safety or the *in vivo* process of the study drugs, including cardiovascular, hepatic, renal, gastrointestinal, endocrine or immune system. (iii) a history of any bleeding disorders. (iv) diseases of nervous system or muscle diseases, that might affect subjects compliance; (v) alcohol or coffee addiction; (vi) participated in another clinical trial or blood donation in previous 2 months; (vii) took any drug treatment within 2 weeks.

### Study design

**Figure 2:**
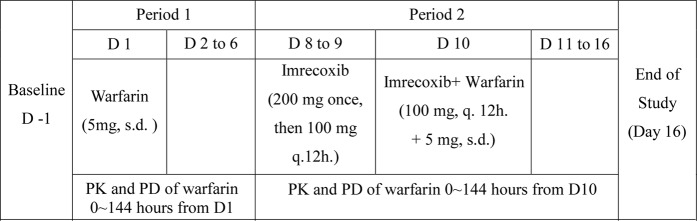
Study design s.d. = single dose.

Current study is phase I clinical trial, which was designed as open-labeled and fixed-sequence, and all the information/data were collected from a single center. This study contained two phases (Fig. 2). In phase one, the volunteers received a 5 mg dose of warfarin alone at 8:00 a.m. on day 1. In the other phase, they orally took imrecoxib to steady-state (200 mg imrecoxib at 8:00 a.m. on day 8, and a 100 mg dose q.12 hours from day 8 to 10, 6 times in total), followed by a 5 mg dosage of warfarin co-administered at 8:00 a.m. on day 10. The volunteers were hospitalized on day-1 (the day before the study), 10 hours of fasting was required before administration^27^. Subjects should avoid any activities involved in risks of haemorrhage^9^. Blood samples (4 mL each) for analysis of pharmacokinetic parameters were obtained 60 minutes before dose of warfarin and 0.5, 1, 2, 3, 4, 5, 6, 8, 12, 24, 36, 48, 72, 96, 120 and 144 hours after dosing. The pharmacodynamics properties of warfarin were expressed by INR and detected by PT before and after 6, 12, 24, 36, 48, 72, 96, 120, 144 hours of warfarin dose^27,28^.

### Analytic methods

A stable LC-MS/MS method was established for detecting S- and R-warfarin plasma concentrations. The chromatographic separation was carried out on an LC system (Shimadzu LC-20AD, Tokyo, Japan) using water and acetonitrile, and AB QTRAP 4000 system (AB Sciex, Foster City, CA, USA) in positive electrospray ion mode was hired for quantification^29–31^. Warfarin-d5 was used as the internal standard. Liquid-liquid extraction with 3 mL dichloromethane: diethyl ether: (2:3, v/v) was employed for 200 μL human plasma. Good linearity was obtained between 5.00–1000 ng/ml for each enantiomer^32^. The inter- and intra- precision (CVs% for 10, 100 and 800 ng/ml) were ≤5.2% for R-warfarin and ≤5.0% for S-warfarin, respectively. Inaccuracy for R-warfarin was between −6.4% to +4.2%, and ranged from −5.9% to +5.1% for S-warfarin. The mean absolute recovery was ≥87.3% (CVs <6.0%)^27,33^.

### Pharmacokinetics and pharmacodynamics analysis

As our previous studies reported^27^, pharmacokinetic analysis was performed base on plasma concentrations of warfarin enantiomers at each time-point by hiring Drug and Statistics Software version 3.1.5. The measurement outcomes contained area under the profile (AUC_0-t_), the terminal half-life (t_1/2_), maximum plasma concentration observed (C_max_), time of maximum concentration (T_max_). AUC from 0 to infinity (AUC_0-∞_). Parameters of pharmacodynamic were estimated from the INR data on each period. PT (INR) was measured with the use of prothrombin complex assay (STA-R, SPA 50 Reagent, Diagnostica stago)^34^. Maximum INR (INR_max_) and baseline INR (INR_baseline_) were determined by PT_test_ divide PT_normal_. The linear/logarithmic trapezoidal method was used for calculation of area under INR-time profile (AUC_0–144h, INR_)^28^.

### Safety evaluations

The safety assessments were conducted on account of clinical examinations, such as evaluation of general subject appearance, vital signs and routine hematology and biochemistry assays^35^, together with adverse events evaluation (AEs), conducted at screening, pretreatment, post-treatment (day 7) and end of trial (day 16). Signs and symptoms relate to study drugs, such as nausea, diarrhea, vomiting, headache and dizziness, were observed and documented by the study physicians^36^. AEs were defined as mild, moderate or severe^37^. Determination of causal relationship between AEs and study drugs followed the criterions announced by the World Health Organization^27^.

### Statistical methods

EpiData 3.0 software was used for data entry and management, statistical analysis was conducted on SAS 9.3 software programming (SAS Institute Inc., Cary, NC). The statistical significance was accepted with two-sided p < 0.05^38^. Pharmacokinetic and pharmacodynamic analyses were based on the subjects who finished trial without great program violation which have a major impact on pharmacokinetic and pharmacodynamic parameters. Descriptive statistics such as mean, median, range, and standard deviation were calculated for observed variables.

Log-transformation of pharmacodynamic parameters INR_max_ and INR_(AUC)_ were applied. Comparing the difference between warfarin treatment and combination treatment for T_max_, logINR_max_ and logINR _(AUC)_ used the F test in ANOVA analysis. The GMR and 90%CIs were calculated by back-transforming for AUC_0-∞_, AUC_0–144h_, C_max_, INR_max_, T_max_ and INR_(AUC)_. The 90% CIs felled within the acceptance range of 0.80–1.25 suggest a lack of drugs interaction^28^.

## Results

### Study population

12 healthy volunteers with CYP2C9*3 AA and VKORC1 homozygous AA genotypes were enrolled. Table 1 shows the demographic characteristics, PT and INR value of the volunteers. No striking differences (p > 0.05) in age, height, weight, PT or INR were observed. Both prothrombin time and INR levels were within normal limits. Since the polymorphisms of CYP2C9 and VKORC1 account for 35–40% anticoagulant efficiency of warfarin, we tested these genotypes of the volunteers. There is no volunteer dropped out from the trial. No volunteers dropped out from the trial.

**Table 1:** Demographic characteristics.

Characteristics	Mean (SD) (n = 12)
Age, years	24.3 (2.2)
Gender, male	12
Ethnicity, Han/minority	11/1
Height, m	1.74 (0.04)
Weight, kg	64.3 (4.7)
BMI, kg/m^2^	21.1 (1.3)
Prothrombin time, s	12.9 (0.6)
INR	1.0 (0.06)
CYP2C9 *3 genotype, **n**	
AA	12
AC	0
CC	0
**VKORC1 genotype**, **n**	
AA	12
AG	0
GG	0

### Safety and tolerability

Both warfarin alone and concomitantly with imrecoxib have safey and good tolerance in healthy volunteers across the trial. Neither severe AEs nor accidental bleeding events occurred during the trial. All the data or information of physical examination, vital signs, laboratory test results or 12-lead ECG were not meaningful altered compare to before administration. In period 1, one subject had transient elevated direct bilirubin (9.6 μmol/L, upper limit of normal = 6.8 μmol/L) on day 7, which met the definition of grade 1 AEs (>ULN — 1.5 × ULN in direct bilirubin). However, the subject did not have any associated signs or symptoms and the level of direct bilirubin stayed normal on day 16 (period 2). Therefore, the investigator considered it to be unrelated to study drugs. In period 2, one subject was observed to be experiencing mild abdominal/upper abdominal discomfort on Day 8 after the first dose of imrecoxib, which continued for about 1.5 hours and disappeared without medical treatment. This event was regarded as possibly related to the drugs. No volunteer dropped out from the trial due to adverse experiences.

### Pharmacokinetics

**Figure 3:**
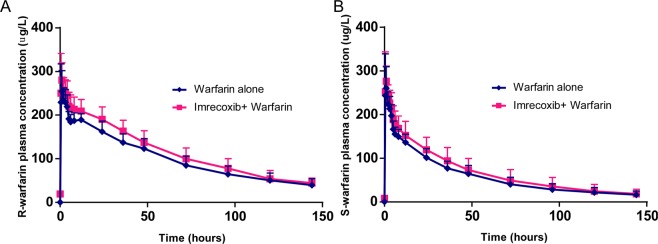
Plasma concentration–time curves of warfarin enantiomers (n = 12).

The pharmacokinetic parameters and pharmacokinetic curves of warfarin enantiomer both warfarin alone and concomitantly with imrecoxib are listed below (Table 2, Fig. 3). Concomitant administration of imrecoxib and warfarin did not change the median T_max_ value of R- and S-warfarin 0.8 (0.5~2.0) hours compared to 1.0 (0.5~3.0) hours with administration of warfarin alone (*P* > 0.300). The t_1⁄2_ of R–warfarin for recipients of warfarin alone and recipients of co-administration of imrecoxib and warfarin were 64.08 ± 15.97 hours and 59.02 ± 9.39 hours, respectively (*P* > 0.1). In the absence and presence of imrecoxib, the t_1⁄2_ for S-warfarin were 57.00 ± 15.27 hours and 51.63 ± 7.59 hours respectively (*P* > 0.1). Receiving imrecoxib did not change V_z_/F of R-warfarin, however, a decrease of 16% was observed for V_z_/F of S-warfarin, the mean V_z_/F slightly decreased from 12.65 ± 2.55 to 10.61 ± 1.89 (*P* = 0.01 for co-administration of imrecoxib versus warfarin alone treatment). As summarized in Table 3, compare imrecoxib and warfarin in combination to warfarin alone, the GMR of R-warfarin AUC_0–144h_ and C_max_ were 1.14 and 1.06, respectively, and the 90% CIs ranged from 0.93–1.13 and 0.98–1.15, both of which were within 0.8–1.25. For the S-warfarin enantiomer, the GMR of C_max_ and AUC_0-t_ were 1.03 and 1.14, and the corresponding 90% CI were 0.93–1.13 and 1.09–1.20. All 90% CIs were in the range of 0.80–1.25. These results suggest that the pharmacokinetic profiles of S- and R-warfarin were not significantly impacted by co-administration of imrecoxib.

**Table 2:** Summary of main pharmacokinetic parameters of warfarin enantiomers.

Parameter	Warfarin + Imrecoxib	Warfarin alone	GMR
**R-warfarin**			
AUC_0-t_ (μg·hr/ml)	16722.49 (3146.74)	14604.16 (2606.56)	1.14
AUC_0-∞_ (μg·hr/ml)	20587.34 (4630.96)	18489.12 (4701.71)	1.12
MRT_0-∞_ (hr)	83.44 (13.39)	88.41 (19.96)	0.96
λz (1/hr)	0.012 (0.002)	0.012 (0.004)	1.09
t_1/2z_ (hr)	59.02 (9.39)	64.08 (15.97)	0.94
T_max_ (hr)	0.8 (0.5–2.0)	1.0 (0.5–3.0)	0.89
Vz/F (L)	10.61 (1.89)	12.65 (2.55)	0.84
CL/F (L/hr)	0.128 (0.030)	0.143 (0.035)	0.89
C_max_ (μg/L)	300.75 (36.61)	285.25 (39.66)	1.06
**S-warfarin**			
AUC_0-t_ (μg·hr/ml)	9869.17 (3214.71)	8529.08 (2124.37)	1.14
AUC_0-∞_ (μg·hr/ml)	11365.73 (4363.24)	9939.63 (3063.76)	1.13
MRT_0-∞_ (hr)	63.35 (12.78)	66.44 (15.76)	0.96
λz (1/hr)	0.014 (0.002)	0.013 (0.004)	1.08
t_1/2z_ (hr)	51.63 (7.59)	57 (15.27)	0.93
T_max_ (hr)	0.8 (0.5–2.0)	1.0 (0.5–3.0)	0.88
Vz/F (L)	17.63 (4.43)	21.76 (7.61)	0.83
CL/F (L/hr)	0.241 (0.060)	0.269 (0.062)	0.89
C_max_ (μg/L)	300.42 (34.01)	294.92 (50.35)	1.03

**Table 3:** Statistical analysis results (GMRs and 90% CIs) of warfarin enantiomers.

parameters	R-warfarin		S-warfarin	
	GMR	90% CIs	GMR	90% CIs
AUC_0-∞_	1.12	1.08–1.16	1.13	1.07–1.18
AUC_0-t_	1.14	1.11–1.17	1.14	1.09–1.20
C_max_	1.06	0.98–1.15	1.03	0.93–1.13

### Pharmacodynamics

**Figure 4:**
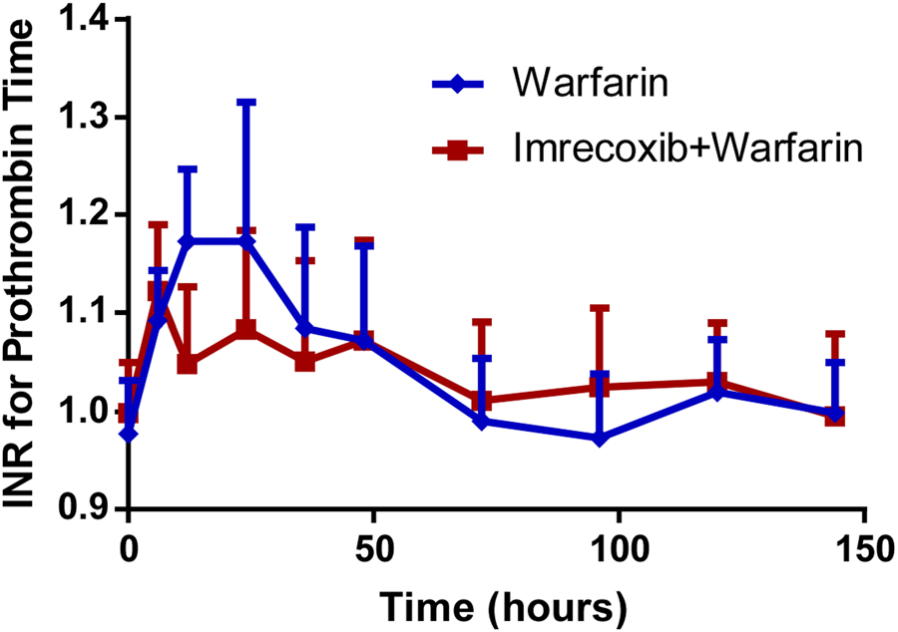
INR -time profiles (n = 12).

The mean INR-time profiles of warfarin alone or concomitant with imrecoxib are shown in Fig. 4. The median T_max_ (time to maximum observed effect) value for warfarin alone and co-administration of warfarin with imrecoxib were 15.10 hours and 14.45 hours, respectively. Although co-administration of warfarin and imrecoxib caused a small, transient decrease in INR value at 12 hours, mean INR values over time were similar between these two groups. The geometric mean ratio of pharmacodynamic parameters (INR_max_, T_max_, INR (AUC), 90% CI, imrecoxib plus warfarin versus warfarin alone) were 0.94 (0.90–0.98), 0.96 (0.92–0.99) and 1.00 (0.99–1.01), respectively. The corresponding 90% CIs for each of these values were entirely within 0.8–1.25 (Table 4). A log transformation was applied for INR_max_ and INR(AUC). There was no significant difference for log INR_max_, logINR(AUC) and T_max_ during concurrent imrecoxib treatment compared with warfarin alone treatment (Table 5).

**Table 4:** Pharmacodynamic Derived Parameters of Warfarin.

parameter	Geometric Mean Ratios (warfarin + imrecoxib: warfarin alone)				
	Warfarin alone	Warfarin + imrecoxib	GMR	90% CI inner bound	90% CI outer bound
INR_max_	1.21	1.14	0.94	0.90	0.98
T_max_	15.07	14.40	0.96	0.92	0.99
INR (AUC)	143.47	142.86	1.00	0.99	1.01

**Table 5:** Statistical analysis results of PT and INR.

parameter		Warfarin alone	Warfarin + Imrecoxib	P value
logINR_max_	n	12	12	F = 2.9841, p = 0.0981
	Mean (SD)	0.191 (0.0988)	0.130 (0.0711)	
	Median	0.195	0.135	
	Min, Max	0.04, 0.38	−0.03, 0.22	
T_max_	n	12	12	F = 2.9147, p = 0.1019
	Mean (SD)	15.11 (1.161)	14.42 (0.788)	
	Median	15.10	14.45	
	Min, Max	13.4, 17.4	12.7, 15.5	
logINR	n	12	12	F = 0.0241, p = 0.8781
(AUC)	Mean (SD)	4.966 (0.0630)	4.962 (0.0718)	
	Median	4.953	4.952	
	Min, Max	4.88, 5.08	4.85, 5.06	

## Discussion

This study revealed the pharmacodynamics and pharmacokinetics of warfarin would not be altered by concomitant administration of imrecoxib with the clinically recommended dosage. As an innovative and mild selective COX-2 inhibitor, imrecoxib can probably be prescribed to patients with cardiovascular disease and stable long-term warfarin therapy^4^. Several studies indicated an increasing INR value of healthy volunteers and accidental bleeding in patients stable on warfarin therapy after dosing celecoxib, which with similar therapeutic efficacy and side effects to imrecoxib^39–43^. Monitoring the INR of long-term warfarin recipients is required to optimize effective dosage because of a large inter-individual variation and narrow therapeutic window^44^. S-warfarin is metabolized by CYP2C9 enzyme^8,22,45^, as well as imrecoxib. S-warfarin directly inhibits vitamin K-dependent coagulation factors^46^, and accounts for 85% anticoagulant activity of warfarin^22^. Competition of the CYP2C9 metabolic enzyme may occur when patients receive warfarin and imrecoxib, which prevents S-warfarin from being metabolized to S-7-hydroxywarfarin, resulting in an increase of plasma concentration and anticoagulant effects of S-warfarin. Both warfarin (99%) and imrecoxib (96%) are highly protein bound in plasma. Imrecoxib may competitively displace warfarin from the protein-binding sites, enhancing blood concentration of free warfarin and increasing bleeding risks. Thus, we speculated that imrecoxib and warfarin may interact.

Inconsistent with our speculation, the results indicated the pharmacokinetic profiles of warfarin enantiomers were not significantly changed by co-administration of imrecoxib. Comparing co-administered warfarin and imrecoxib with warfarin alone, for S-warfarin, the outer bound of 90% CIs of AUC_0–144h_ increased to 20% (Table 3), but AUC_0–144h_ and AUC_0-∞_ were not significantly changed (Table 2), and the GMRs for and 90% CIs for AUC_0-∞_, AUC_0–144h_ and C_max_ were all within 0.80–1.25 (Table 3). PT were expressed by an INR value in this study. Monitoring of PT is required for individualized dosage adjustments in clinical warfarin use. There were no meaningful disparities in T_max_, logINR _(AUC)_ and logINR_max_ observed between two treatments. Although the mean INR at 12 hour, near the T_max_, was significantly reduced when dosed with imrecoxib, the GMR and 90% CI of INR AUC_0–144 h_ for warfarin + imrecoxib: warfarin only were near identical, 1 (0.99, 1.01) (Table 4), and no significant difference in logINR_max_ was observed (Table 5). These results suggested imrecoxib would not alter the pharmacokinetics parameters and anticoagulation activity of warfarin, but greater caution should be taken in the wider applicability of the results.

It has been widely agreed that the anticoagulant efficiency of warfarin is highly related to genetic polymorphisms. Among these genes, CYP2C9 and VKORC1 are responsible for 30% to 40% of the warfarin efficiency differentiation^47–51^. People with these polymorphisms show a significant difference in warfarin pharmacodynamic and pharmacokinetic profiles compared to wildtype subjects. For a better evaluation, all volunteers enrolled in this study were CYP2C9*3 AA genotype and VKORC1 (G-1639A) with homozygous AA genotype. Several studies have reported the frequency of CYP2C9 *3 AA and AC genotypes were 95% and 5%, respectively, and mutation frequency of VKORC1–1639 AG and AA were 7.4% and 92.6% in the Han-Chinese population^52–57^. Warfarin-induced hemorrhage associated with age^58^. Significant reduction in clearance of warfarin with age was also reported^59^. Healthy volunteers aged from 18 to 45 years old are recommended by guideline^26^. However, in a large Japanese reports analysis, the reporting odds ratio of hemorrhagic events associated warfarin in patients age 40–49 significantly lower than those aged ≤40 or those aged ≥50^60^. In addition, many studies focusing on age and warfarin’s efficiency divided the volunteers’ age into young and elderly groups. The age range of these groups was 18–40 and 65–90 respectively. Therefore, we enrolled the volunteers between 18 and 40 years old to rule out the influence of age on warfarin.

A loading dose of 200 mg imrecoxib was chosen, then subsequently taking 5 continuous 100 mg doses of imrecoxib in order, to guarantee imrecoxib reaches its steady-state concentration prior to warfarin dose in this study. Clinically recommended dosage is 5 mg for warfarin and 100 mg for imrecoxib. The dosage of warfarin used in some studies was 25 mg^61–63^. We used 5 mg warfarin in both periods, to ensure adequate plasma drug levels close to common clinic levels while avoiding exposing participants to unnecessary bleeding risks caused by excessive use of warfarin. Consistent with our study, the existence of an interaction between warfarin (5 mg/d) and celecoxib was evaluated in a study with 24 healthy volunteers^64–66^, and 7.5 mg warfarin was used to examine potential drug interactions with celecoxib in healthy volunteer studies^28,67^.

In conclusion, this study revealed that co-administration of imrecoxib did not affect the pharmacokinetic parameters or anticoagulant properties of warfarin. Thus, we concluded that adjusting dosage is not necessary when administering imrecoxib concomitantly with warfarin. However, we only conducted a single dose study of warfarin, so the possibility that a higher dosage or multiple doses of warfarin would alter its pharmacokinetic or pharmacodynamic profiles during co-administration with imrecoxib could not be excluded, though the clinical relevance would be doubtful.
